# Categorizing Sequences of Concern by Function To Better Assess Mechanisms of Microbial Pathogenesis

**DOI:** 10.1128/iai.00334-21

**Published:** 2022-03-22

**Authors:** Gene D. Godbold, Anthony D. Kappell, Danielle S. LeSassier, Todd J. Treangen, Krista L. Ternus

**Affiliations:** a Signature Science, LLC, Charlottesville, Virginia, USA; b Signature Science, LLC, Austin, Texas, USA; c Department of Computer Science, Rice University, Houston Texas, USA; University of California, Santa Cruz

**Keywords:** biodefense, bioinformatics, biothreat, controlled vocabulary, host-pathogen interactions, immune evasion, microbial pathogenesis, ontology, sequence of concern, sequence screening

## Abstract

To identify sequences with a role in microbial pathogenesis, we assessed the adequacy of their annotation by existing controlled vocabularies and sequence databases. Our goal was to regularize descriptions of microbial pathogenesis for improved integration with bioinformatic applications. Here, we review the challenges of annotating sequences for pathogenic activity. We relate the categorization of more than 2,750 sequences of pathogenic microbes through a controlled vocabulary called Functions of Sequences of Concern (FunSoCs). These allow for an ease of description by both humans and machines. We provide a subset of 220 fully annotated sequences in the supplemental material as examples. The use of this compact (∼30 terms), controlled vocabulary has potential benefits for research in microbial genomics, public health, biosecurity, biosurveillance, and the characterization of new and emerging pathogens.

## INTRODUCTION

## WHAT MAKES “BAD BUGS” BAD?

The “worst” pathogens of humans cause severe disease in those possessing normal immunity. Pathogens of other organisms indirectly affect our species by damaging the livestock or crops on which we depend for sustenance. High-level biological phenotypes of microbes, such as pathogenicity, transmissibility, and environmental stability, are complex (1), but they are products of specific microbial sequences encoded within the parasite genomes. Pathogenicity toward one or more host organisms, transmissibility within a species of host organism or between that host organism and vectors (or natural reservoirs), and stability within a specified environment will not be retained if certain sequences are unexpressed.

Responsible parties have been concerned about engineered biothreats for years (1). The increasing technical prowess of synthetic biologists and the burgeoning business of nucleic acid providers have brought the limitations of existing guidance for assessing risk and the adequacy of screening protocols into sharp relief (2). In the past, the “bad microbe” model assessed threat based on pathogens that could pose a severe threat to public health and safety. The “bad microbe” conception has waned, with the “sequence of concern” (SoC) model taking its place (1).

In evaluating SoCs for their risk to public safety, we discovered a dichotomy in sequence annotation. UniProt has a well-curated set of eukaryotic and bacterial toxins, although the targets of those toxins are not always noted (3). Viral parasitism can also be adequately related with the use of existing gene ontology (GO) terms. However, there are few terms for describing parasitism of hosts as practiced at the molecular level by bacterial, fungal, and protozoal pathogens. What terms there are have few annotations associated with them. Often, the only hint in UniProt that a sequence might be involved in deleterious host-affecting activities was through the tag “GO:0009405 (pathogenesis).” As of June 2021, this term was associated with over 277,000 UniProt accession numbers. Interestingly, the GO:0009405 pathogenesis term has been scheduled for obsolescence, with the final notice given in March 2021 (https://github.com/geneontology/go-annotation/issues/3452).

SoCs are not limited to organisms and toxins on the select agent lists (1). Simply listing the genes of those microbes and toxins would include tens of thousands of innocuous sequences that these parasites share with their close, but nonpathogenic and even nonsymbiotic, relatives (i.e., false positives). This also neglects sequences that cause damage or enable infection from human-disease-causing microbes not deemed serious enough for inclusion on select agent lists (i.e., false negatives). This minireview offers criteria to identify SoCs based on an analysis of more than 2,750 sequences culled from the professional literature for more than 105 bacterial species, 85 viruses, and 25 eukaryotic pathogens. We describe an approach to better characterize these sequences for bioinformatic applications.

## WHAT ORGANISMS ENCODE SEQUENCES OF CONCERN?

Of the hundreds of thousands of species of bacteria, fungi, protozoa, worms, and viruses on the planet, only a small percentage have been documented to cause disease in the primate Homo sapiens. It was estimated in 2007 that ∼1,400 microbes and parasites can produce disease in humans. Of these, 541 were bacterial, 325 fungal, 285 helminthic, 189 viral, and 57 protozoal (4). Further studies indicated that ∼600 fungi can cause disease in humans (5), and well over 200 RNA viruses can infect humans (6), so the total number of human-disease-causing entities is greater than 1,750 and is probably closer to 2,000.

Parasites are distinguished from closely related symbionts by their expression of specific molecules that, when deployed appropriately, can cause a loss of homeostasis (i.e., disease), in a susceptible host. Particular environmental conditions can dispose a host toward greater susceptibility and a parasite toward greater disease-generating ability (7). While many sequences from human-disease-causing microbes have been examined empirically, “the majority…from the microorganisms responsible for the world's most prevalent diseases remain poorly defined and uncharacterized” (8).

## MICROBIAL PATHOGENESIS AND VIRULENCE FACTORS

Practitioners of the biological subspecialty of microbial pathogenesis, a hybrid of cellular biology, molecular biology, and microbiology, investigate the sequences by which microbes exploit host organisms. Perhaps the earliest exploration occurred 50 years ago in swine by Williams Smith and Margaret Linggood. They showed that nonpathogenic Escherichia coli could become an enterotoxigenic pathogen with the introduction of plasmids encoding F4 fimbriae and enterotoxin (9).

Testing a mechanism that directly contributes to pathogenesis makes for the most satisfying investigations. In 2007, experiments were conducted using mice of the same genetic background, while the Citrobacter rodentium bacteria used to infect the mice were varied in which set of up to seven effectors they expressed. The authors showed how the set of sequences expressed rendered the pathogen capable, less capable, or incapable of transmission to a new host and more or less proficient at causing lethal damage (10). Unfortunately, there are more than a few papers declaring a gene product a “virulence factor” after experiments show a “decrease in virulence” following deletion of the gene, though no mechanism can be inferred. In the absence of adequate controls, the gene product in question may simply be necessary to the normal functioning of the organism without necessarily affecting the host.

### (i) When “virulence factors” are not sequences of concern.

The “virulence factor” appellation is rife in the literature. “Factor” covers carbohydrates, lipids, proteins, and combinations thereof, as well as small RNAs. Encoded virulence factors are *prima facie* candidates for SoCs. However, molecules called virulence factors are not always a threat to a host. Bacterial siderophores are called virulence factors, but most are scavenging molecules without which the bacterium would perish in any environment where metal cofactors are rare. It makes more sense to designate these “virulence lifestyle” sequences (11), or perhaps “proliferative factors.” The less-than-discriminating use of “virulence factor” makes it difficult for investigators to discern what sequences actually harm a host (12). Not all virulence factors are SoCs.

Researcher designations of virulence factors are critical for curators to recognize them, but the less-than-thoughtful use of the nomenclature can create problems for bioinformaticians. An analysis of 2,000 purported virulence factors from over 50 bacterial pathogens found that just 620 were specific to pathogens while 1,368 were common to both pathogens and nonpathogens. The 620 pathogen-specific virulence factors were more likely to reside in pathogenicity islands and be secreted via a secretion system (13). In contrast, the 1,368 “common” virulence factors are probably not SoCs. If put into a reference database of “virulence factors,” they would be false positives. An adequate system for categorizing SoCs should recognize these differences.

### (ii) Existing virulence factor data sets and the importance of manual curation of function.

Many databases of virulence factors do not curate their sequences according to an established rubric that allows for the extraction of function. The Virulence Factor Database (VFDB) is limited to bacteria pathogenic for humans. The developers eschew manual curation (14). The data set associated with VFDB includes ∼3,400 sequences from ∼21 bacterial species. No justification is given for the presence of constituent sequences. No curations keyed to individual sequences are provided. The Pathogen-Host Interaction Database (PHI-Base) captures the genetics of pathogen-host interactions from the primary literature along with some functional details, but it principally notes changes in virulence that accompany genetic variants. The effect that these parasite sequences have on the host are of secondary importance (15). The same is true of the Victors database (16). A comparison of bacterium-related databases suggests that functional annotation of SoCs is not a significant concern (17). We think that manual curation is required to adequately annotate the consequences that SoCs have on host processes and enable further advances in computational biology.

## IDENTIFYING AND ASSESSING SEQUENCES OF CONCERN

**FIG 1:**
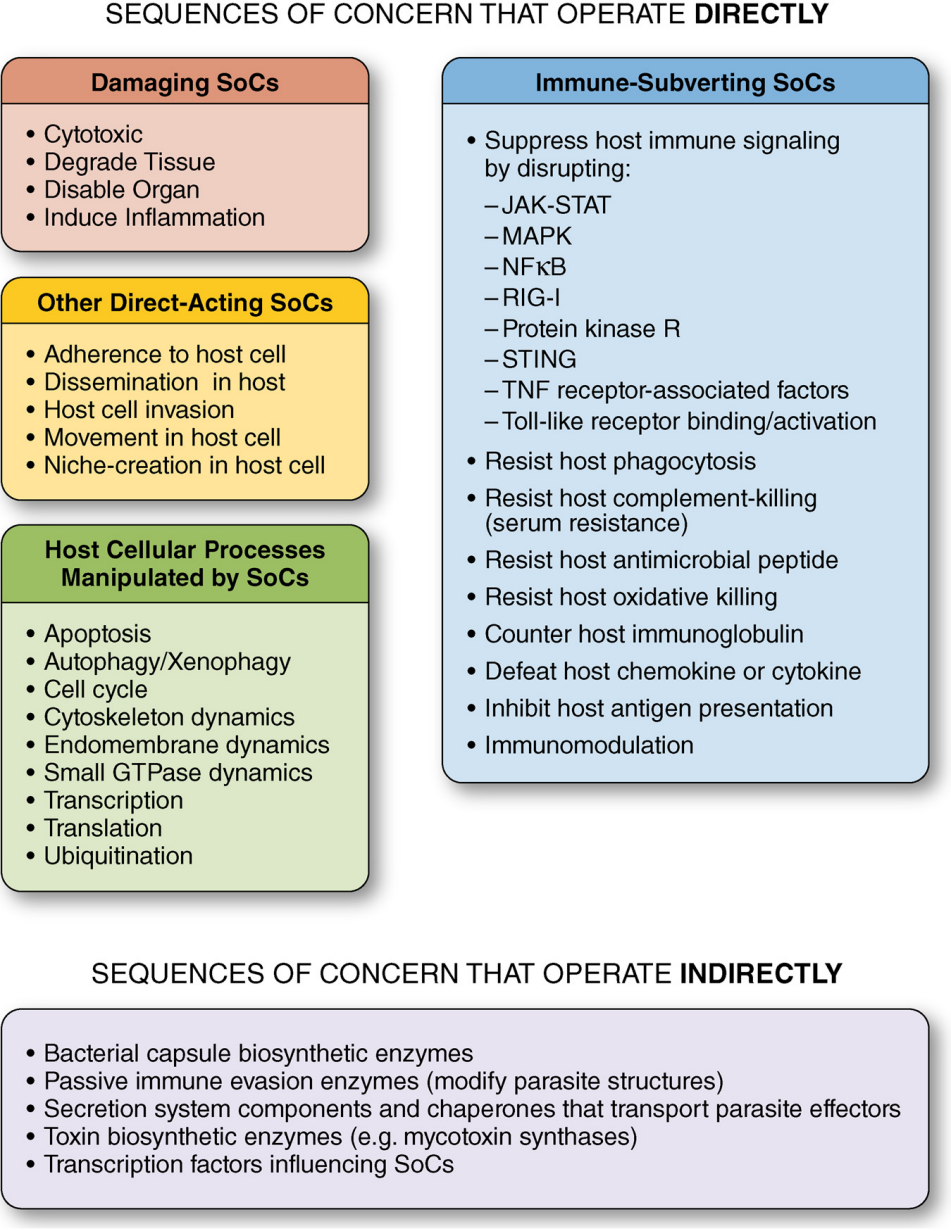
Overview of functions of sequences of concern (FunSoCs) acting directly and indirectly.

There is a chicken-and-egg aspect to identifying SoCs. One must have some idea of what microbial features might be threatening to know what to examine, but it is not until “enough” sequences are perused that the important aspects can be recognized categorically. By reviewing the literature, we discovered sequences that appear important to pathogenesis for parasites of humans, as well as those of animals and plants necessary to human well-being. We have documented over 2,750 of these, which we hope is a fair sample to develop a conceptualization for understanding biothreats. Assessing sequences of concern for their danger in a bioengineering, gain-of-function (GoF) scenario required us to consider two parameters: (i) the effect on the host, including which host processes are manipulated, and (ii) how directly the sequence exerts its effects. For this minireview, we limit ourselves to reviewing functions of SoCs (FunSoCs) from microbes targeting mammals. The FunSoCs are summarized in Fig. 1 and discussed below. Included as supplemental material is a table of short definitions for the FunSoCs (Data Set S1) and a spreadsheet (Data Set S2) with 220 sequence types from 60 pathogenic species (bacterial, fungal, protozoal, viral) annotated with UniProt accession numbers, FunSoCs, and PubMed identifiers to illustrate our curation.

### (i) What is the effect of the SoC on the host?

*(a) Host damage as the* sine qua non *of pathogenicity.* It is generally true that lethal infections are deadly because one or more organs become disabled from cumulative damage. Ascertaining the proximal cause of damage can be problematic. Host damage can be the direct result of the parasite’s action on the host, the host’s reaction to the parasite, or both. While infectious disease theorists of the 20th century once credited the pathogen with unique disease-causing ability, this is no longer tenable (18–20).

Damage is the hallmark of pathogenicity (21). Since this is the case, “toxins” might be said to occupy the preeminent place among virulence factors since they are among the most damaging of molecules deployed by pathogens. In bacteria, toxins are distinguished from damaging effectors in that the former are capable of mediating their own attachment and invasion into a cell, while effectors must be secreted (22).

The term “toxin” is notably nonspecific and amounts to little more than a verbal tag that a molecule is inimical to the life of one or more taxa. But the taxa susceptible to the toxin need to be understood. Alpha-amanitin, bicuculline, carbon monoxide, chlorine gas, ciguatoxin, cyanide, MARTX from *Vibrios*, ricin, and sarin have disparate modes of action and are all deadly to mammals if administered appropriately. In contrast, the toxins of toxin/antitoxin (TA) systems are not hazardous for mammals; however, they might be administered (23). Of course, toxins do not exhaust the range of damaging biological sequences. The following paragraphs attempt to categorize host damage caused by SoCs.

1. Some SoCs lead to direct killing of a cell by enzymatically compromising a vital process (like translation) or by perforating the membrane via a pore-forming protein or a destabilizing enzymatic process. This includes disabling a cell, as with Shiga toxin from *Shigella*/Escherichia coli (24), or membrane destabilization, as with candidalysin from Candida albicans (25, 26). The tripartite HBL enterotoxin from Bacillus cereus (27–29), beta toxin from Clostridium perfringens (30, 31), leukotoxin from Aggregatibacter actinomycetemcomitans (32, 33), VCC from Vibrio cholerae (34), VopT (35) and VopV (36) from Vibrio parahaemolyticus, and phospholipase A2 from Vibrio vulnificus (37) are other such SoCs.
2. Degrading a tissue can be accomplished by proteolysis of the extracellular matrix, loosening the attachments between cells, or liberating a cell from a tissue. The last is sometimes called the “cytopathic effect.” SoCs that accomplish this include aerolysin from Aeromonas hydrophila (38), InhA from Bacillus anthracis (39), fragilysin from Bacteroides fragilis (40), HtrA from Campylobacter jejuni (41), candidalysin from C. albicans (42), NS1 from dengue virus (43), secreted autotransporter toxin (44), the plasmid-encoded toxin (45), cell cycle-inhibiting factor (46), cytolysin A (47), NleA, Map, and EspF (48) from E. coli, CagA from Helicobacter pylori (49, 50), Mip from Legionella pneumophila (51), collagenase A from *Leptospira* (52), Alp1 from Neosartorya fumigata (53), MIF from Plasmodium berghei (54), exfoliative toxins A and B from S. aureus (55), and VopF from V. cholerae (56).
3. Disabling an organ system is the severest type of damage. SoCs that accomplish this include ExoU (57) and ExlA (58) from Pseudomonas aeruginosa, CARDS toxin from Mycoplasma pneumoniae (59), epsilon toxin (60) and iota toxin (61) from C. perfringens, edema toxin (62) and lethal toxin (63, 64) from B. anthracis, cholera toxin from V. cholerae (65), pneumolysin from Streptococcus pneumoniae (66), TcdA and TcdB from Clostridioides difficile (67), lethal toxin from Paeniclostridium sordellii (68, 69), staphylococcal and streptococcal superantigens (70, 71), and NSP4 from rotavirus, a rare example of a viral toxin for mammals (72, 73).
4. SoCs in the class that specifically instigate a damaging inflammatory response appear to directly interact with host components to provoke an inflammatory reaction. These include alpha-hemolysin from Staphylococcus aureus (74, 75), PE11 (76) and PE_PGRS17 (77) from Mycobacterium tuberculosis, Loa22 from Leptospira interrogans (78), pertussis toxin from Bordetella pertussis (79, 80), SipA from Salmonella (81, 82), ExhC from Staphylococcus sciuri (83, 84), Nhha from Neisseria meningitidis (85), GRA24 from Toxoplasma gondii (86), VvpM from Vibrio vulnificus (87), and nucleocapsid protein (88, 89), spike glycoprotein (90, 91), membrane protein (92), ORF3a (93), and Nsp1 (90) from SARS-CoV. Induction of inflammation can be hard to differentiate from the host reaction to microbial provocation that results in inflammasome activation (94). This class of effectors may require splitting into microbially induced versus microbially provoked host inflammation.

*(b) Immune subversion as an essential condition for pathogenicity.* Stanley Falkow observed that the avoidance of host defense mechanisms was a feature of disease-causing bacteria (95). Sequences that subvert innate immune pathways are also found in fungal and protozoan parasites and are a universal feature of viruses. Immune systems embody the “wisdom” of hundreds of millions of years of adaptation over which they have had to detect, deflect, and defeat micro- and macroparasites (96–98). More than 6% of all human genes have a role in immunity (99). Immune systems impose layers of molecular and cellular obstacles to thwart invaders that breach epidermal barriers. Parasites survive these host stratagems by employing molecules that mask their presence, mimic and/or misdirect host responses, or simply eliminate immune effectors. Of the SoCs that we documented, ∼60% of the viral sequences and ∼20% of the bacterial and eukaryotic sequences subvert host immune responses.

Deficits in immune detectors and effectors of a host can render commensal symbionts pathogenic and infections with “nuisance” organisms lethal. Subtle changes in the sequence of a single host immune effector molecule can mean the difference between life and death during challenge with a parasite (100). The study of human immune deficiencies shows the critical importance of these components of innate immunity for defense against the specific, usually narrow, set of parasites against which they defend (101–103). Many infections run their nonlethal course according to the life cycle of the parasite when facing an average host immune response. These are sometimes called “self-limiting” infections, but a defect or deficit in a host immune component can abolish the limitation and produce a life-threatening disease.

**FIG 2:**
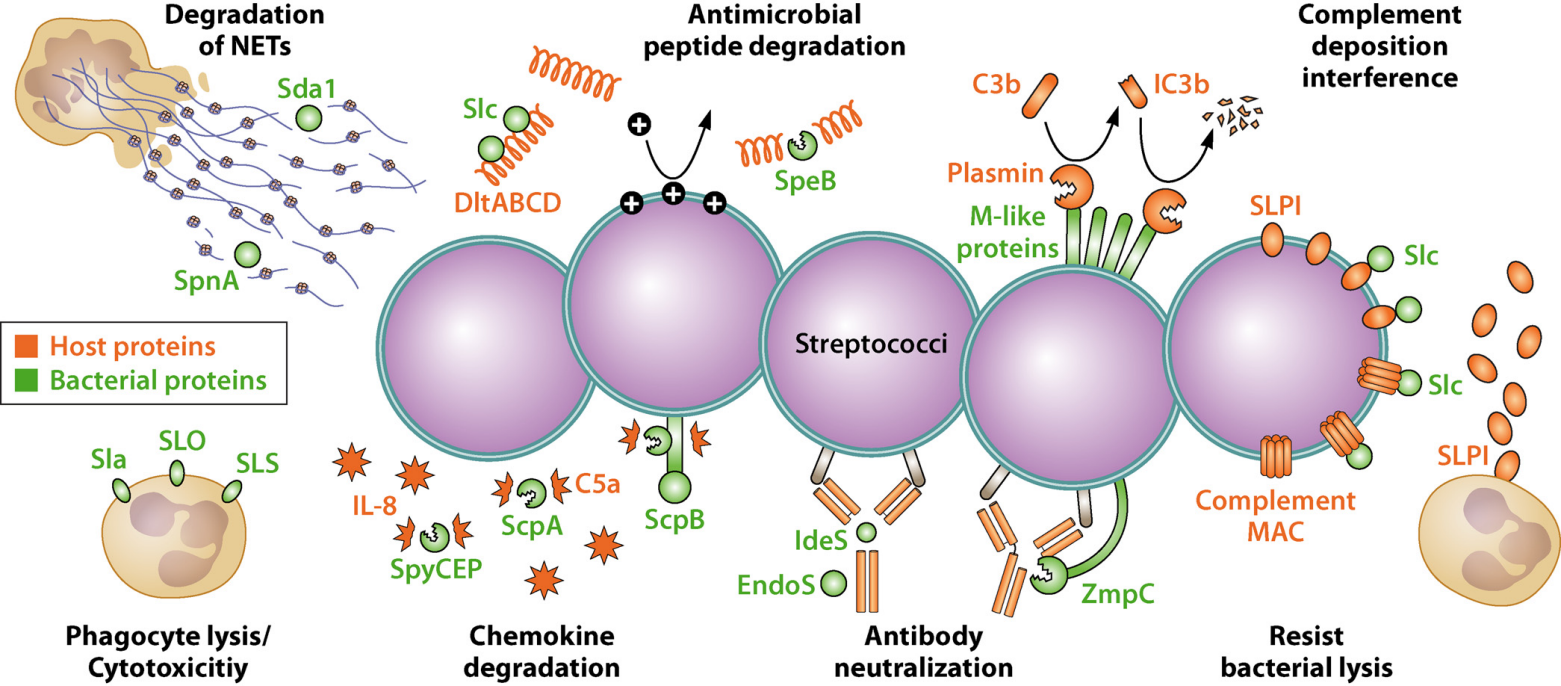
Examples of immune subversion by streptococcal effectors. Host phagocytes are debilitated by streptolysin O (SLO) (70), streptolysin S (SLS) (294, 295), and secreted phospholipase A2 (Sla) (296). Neutrophil extracellular traps (NETs) are countered by the Sda1 and SpnA nucleases (264, 265). Antimicrobial peptides are inactivated by the secreted streptococcal inhibitor of complement (Sic) and SpeB proteases (200, 201). M-like proteins bind host factor H and plasminogen/plasmin, which inactivate host complement components to protect the bacterium (297). Sic protects streptococci from phagocytosis by neutrophils, resists the host complement membrane attack complex (MAC) (70), and counters the antibacterial actions of the host secretory leukocyte proteinase inhibitor (SLPI) (200, 201). Host antibodies are destroyed by membrane-associated ZmpC (226) and the secreted IdeS proteases (222) and inactivated by sugar-cleaving EndoS (223). The group B Streptococcus C5a peptidase ScpB is a serine protease and surface invasin (298) that reduces the neutrophil response and bacterial clearance by cutting the chemoattractant C5a (299). The streptococcal complement protector ScpA helps the bacterium resist phagocytosis (183) and also inactivates C5a (300). SpyCEP eliminates the neutrophil chemoattractant IL-8 (230) and other chemokines (225). Note that this figure depicts SoCs found in both group A and group B streptococci for illustrative purposes, but they would not naturally occur together.

Of the ∼2,000 parasites that can cause disease in humans, the majority are opportunistic: limited to infecting immunocompromised persons (4, 5). The “opportunity” occurs when a proto-parasite encounters an individual whose immune defenses are diminished from (i) loss of barrier function, (ii) congenital immune defects, (iii) infection with HIV, (iv) immune-suppressing pharmacotherapy, or (v) other disease states that alter the homeostasis of the host. These render the host susceptible to microbial parasites that could not successfully establish themselves otherwise. SoCs mediating immune subversion essentially make a host susceptible in the absence of a compromised immune system. Some immune-evading SoCs from Streptococcus are shown in Fig. 2.

1. **Suppression of host immune signaling**. SoCs that subvert the immune system by disrupting host immune signaling comprise a large set; some subdivisions are listed below.
    a. **Disruption of host mitogen-activated protein kinase signaling.** Some SoCs work by directly interfering with a component of the host’s mitogen-activated protein kinase signaling pathways (e.g., p38MAPK, JNK, ERK1/2) or a molecule proximal to them. For example, NleD (104) and NleL (105) of E. coli, SptP (106, 107) and SpvC (108, 109) from Salmonella, OspF from *Shigella* (108, 110), vaginolysin from Gardnerella vaginalis (111), GRA24 from Toxoplasma gondii (86), YopJ from *Yersinia* (112), and M2L from vaccinia virus (113).
    b. **Inhibition of host NF-κB activation.** Some SoCs affect IkappaB, RelA, p50, IKK, NEMO, or a molecular constituent proximal to them, for example, AexU from Aeromonas hydrophila (114), BopN from *Bordetella* (115, 116), TssM from Burkholderia pseudomallei (117), AvrA (118) and GtgA (119) from Salmonella, InlC from *Listeria* (120), IpaH1.4, IpaH2.5 (121), IpaH9.8 (122, 123), and OspG from *Shigella* (124, 125), NleC (126, 127), NleE (128), and NleH1/2 (129, 130) from E. coli, MavC from L. pneumophila (131), and BPLF1 from Epstein-Barr virus (132).
    c. **Manipulation of host signaling through tumor necrosis factor (TNF) receptor-associated factor (TRAF)**. SoCs can manipulate sequences downstream of the TNF receptor and upstream of NF-κB, for example, TssM from B. pseudomallei (117), NleB from Citrobacter rodentium (128, 133, 134), BPLF1 (132) and LMP1/BNLF1 (135) from Epstein-Barr virus, NleB1 (128, 136) and Tir (137, 138) from E. coli, SseK1 from Salmonella (128, 139), OspI from *Shigella* (140), GRA7 (141) and GRA15 (142) from T. gondii, K7R from vaccinia virus (143, 144), and YopJ from Yersinia pestis (145).
    d. **Disruption of signaling from host Toll-like receptors**. Disruption of signaling from Toll-like receptors can occur through alteration of the abundance of the host ligand or receptor, alteration of the ability of the ligand to bind to the receptor, or direct agonism/antagonism of the host receptor or cellular cofactors. SoCs engaging in these activities include PI-PLC from B. anthracis (146), envelope glycoprotein from Ebola virus (147), BGLF5 from Epstein-Barr virus (148), PE9-PE10 from M. tuberculosis (149), and Ssl3 (150) and Ssl4 (151) from S. aureus.
    e. **Disruption of host JAK-STAT signaling**. Many viral proteins, including NSP2 from Chikungunya virus (152) and ORF6 from severe acute respiratory syndrome coronavirus (SARS-CoV) (153), target the JAK-STAT signaling pathway for antiviral defense.
    f. **Disruption of host RIG-1 signaling**. Keeping RIG-1 inactive through sequestration or targeted destruction of RIG-I or proteins immediately proximal to it via ubiquitination is a function of many viral proteins. The 3C proteinase of human poliovirus cuts host RIG-1 to prevent interferon activation (154).
    g. **Disruption of host protein kinase R activity**. The disruption of host protein kinase R activity can occur by sequestering viral double-stranded RNA (dsRNA), by manipulating the phosphorylation of host elongation factor 2-alpha, and by directly binding host PKR. E3L of vaccinia virus binds viral dsRNA to prevent it from activating of host protein kinase R and OAS (155). NS1 from influenza virus (156) and VP35 from Marburg virus (157) also attenuate antiviral signaling.
    h. **Inhibition of host STING activity**. Both E1A from human adenovirus and E7 from papillomavirus inhibit the cGAS-STING pathway, along with many other viral proteins (158).
2. **Resistance to phagocytosis**. SoCs interfering with host phagocytosis of microparasites act through a variety of mechanisms, including inhibiting opsonization, manipulating the cytoskeletal dynamics of host phagocytes, and antagonizing phagocyte receptors. These SoCs include AexU from A. hydrophila (159), BadA from Bartonella henselae (160), AC toxin (161) and BteA (162) from *Bordetella*, OspB from Borrelia burgdorferi (163), Hgt1p from Candida albicans (164), App1 from Cryptococcus (165, 166), GelE from Enterococcus faecium (167), EspJ (168) and Pic (169) from E. coli, RodA from *Neosartorya fumigata* (170), ExoS (171) and ExoT (172, 173) from P. aeruginosa, aureolysin (174), CHIPS (175), Efb (176, 177), Sbi (178), SCIN (179), and Spa (179, 180) from S. aureus, BibA (181), M protein (182), ScpA (183), and Sic (70) from Streptococcus, RtxA from V. vulnificus (184), VopQ from V. parahaemolyticus (185), and PsaA (186), YopE (187), YopH, YopO/YpkA, and YopT (188–190) from *Yersinia*.
3. **Resistance to complement-mediated killing**. Host complement effectors can be directly proteolyzed, as by Vag8 of B. pertussis (191), or inactivated indirectly, as by CipA from Acinetobacter baumannii, which recruits host plasminogen to the bacterial surface (192). BclA of B. anthracis mediates serum resistance by recruiting factor H, a host complement control protein, to the bacterial surface (193).
4. **Resistance to antimicrobial peptides**. Host antimicrobial proteins are cationic peptides that interact with the negatively charged bacterial membrane. They can be destroyed by bacterial proteases, including OmpA from Klebsiella (194), ClpX from B. anthracis (195), CPAF from Chlamydia (196), staphylokinase from S. aureus (179), SepA from Staphylococcus epidermidis (197), DRS (198), SspA, SspB (199), SpeB, and Sic from Streptococcus (200, 201), and OmpU from V. cholerae (202).
5. **Resistance to oxidative killing**. Host oxidases can be neutralized by bacterial effector molecules, including superoxide dismutase from B. anthracis (203), SodC from Coxiella burnetii (204), KatN from E. coli (205), SodC from Francisella tularensis (206), and SOK from S. aureus (207). The generation of reactive oxygen (or nitrogen) species can also be countered by upstream legerdemain, as with EtpE from Ehrlichia chaffeensis (208), Ndk (209), PPE2 (210), PE5, PE15 (211), and PE_PGRS62 (212) from M. tuberculosis, SopB from Salmonella (213), VopL from V. parahaemolyticus (214), and YopH from Y. pestis (188, 215).
6. **Countering immunoglobulin**. Parasite effectors can sequester, destroy, or neutralize immunoglobulins by other means, as exemplified by BatB from *Bordetella* (216), IgA1P from Haemophilus influenzae (217), IbpA from Histophilus somni (218, 219), Sbi (178), Ssl7 (220), Spa (180), and staphylokinase (221) from S. aureus, IdeS (222), EndoS (223), SibA (224, 225), and ZmpC (226) from Streptococcus, and InvD from Y. pseudotuberculosis (227).
7. **Defeat of cytokines**. Pertussis toxin from B. pertussis, Lpd from P. aeruginosa, CHIPS, Eap, FPRL1 inhibitory protein, and Ssl5 (228) from S. aureus, PrpL (229) and SpyCEP (230) from Streptococcus, BARF1 from human herpesvirus 4 (HHV-4) (231, 232), and a plethora of orthopoxviral receptors/binding proteins can form associations with host TNF, interleukins, chemokines, and interferons to dysregulate host immune signaling (233–243).
8. **Inhibition of antigen presentation**. Pertussis toxin from *Bordetella* (80), EsxG, EsxH (244), Vpu from HIV-1 (245), ORF66 from HHV-3 (246), BILF1 (247, 248), BNLF2a (249–251), and BZLF1 from HHV-4 (252), E1A and E3 from human adenovirus (253, 254), LpqH (255, 256), LprA (257), LprG (258), and PPE38 (259) from M. tuberculosis, SteD from Salmonella (260), and IpaH4.5 from *Shigella* (261) inhibit host antigen presentation by various mechanisms.
9. **Resistance to other host immune effectors**. As they are expiring, host neutrophils process their nuclear DNA to create neutrophil extracellular traps (NETs) capable of trapping and killing microparasites. Bacterial nucleases that can counter these include Nuc from Neisseria gonorrhoeae (262) and EndA (263), Sda1 (264), and SpnA (265) from Streptococcus.
10. **Immunomodulation.** Immunomodulation occurs when a parasite protein directly affects aspects of the host immune system in a fashion that does not suggest an obvious advantage for the parasite relative to the host. Sequences include Hcp from A. hydrophila (266), AnkX (267) and LegC4 (268, 269) from *Legionella*, EspC (270), Psts1 (256), PE9, and PE10 (149) from M. tuberculosis, and SspH2 from Salmonella (271).

*(c) Adherence to the host cell.* To affect the host, symbionts need to either secrete toxins that act while the microbe is at a distance from the host cell or contact host cells or tissues directly. This requires specific adhesin molecules that anchor them, however durably, to the host. Toxins also require adhesins to recognize target cells. Adherence can be to specific host protein receptors, to carbohydrate moieties of glycoproteins or glycolipids, to membrane cholesterol, and/or to components of the host extracellular matrix. Such proteins are abundant, and host attachment is often just one of their functions (272, 273).

*(d) Dissemination in the host.* Dissemination factors enable the breaching of host barriers. A breach can happen by proteolytic digestion of tissues or the release of junctional adhesins to allow parasite passage. SoCs that degrade tissue can also be dissemination factors. Examples include ExoS and ExoU from P. aeruginosa (274), InhA from B. anthracis (39, 275), and staphylococcal exfoliative toxins (50, 55, 276).

*(e) Host cell invasion.* A microsymbiont can “enter” a host cell easily when the host cell is a professional phagocyte, but this happens under conditions unfavorable for symbiont survival. Invasins mediate microbial entry into a range of host cells, including nonphagocytic ones, in ways that allow the parasite a greater probability of reproductive success. Bacterial toxins also possess invasive subunits that enable their entry into host cells; this distinguishes them from effectors, which require a secretion system (22).

*(f) Movement in host cell.* Movement within a host cell allows a parasite to circumvent host barriers and avoid programmed defenses. Some intracellular bacteria, as well as vaccinia virions, hijack host actin polymerization to propel themselves into adjacent cells. They thus avoid exposure to the hazards of the extracellular milieu (277).

*(g) Niche creation in host cells.* Some cellular microbial symbionts manipulate host cell processes to create intracellular niches, where they are protected from host destruction and in which they replicate. This has been investigated most thoroughly in Brucella, Chlamydia, *Coxiella*, *Ehrlichia*, *Legionella*, *Listeria*, *Mycobacteria*, and Salmonella. SoCs from these bacteria are generally secreted and subvert the normal endosomal and cytoskeletal dynamics of the host cell. Sorting out the mechanisms for these effectors—there are hundreds just in *Legionella*—is exceedingly complicated, as many are redundant (278).

### (ii) How directly does the sequence exert its effect?

When considering the ease with which the disease-causing capacity of a pathogen might be enhanced by sequence addition/gain-of-function (GoF), it is important to consider how directly the SoC acts on the host. SoCs that act independently without the need for extra (i.e., secondary or tertiary) sequences would affect virulence more parsimoniously. There are at least four levels of SoC involvement in pathogenesis.

1. Type 1 sequences that directly interact with host molecules to contribute to disease are the most concerning. The SoCs described above (i.e., damage, immune evasion, adherence, invasion, movement, dissemination, niche creation) act directly to produce a specific deleterious effect.
2. Type 2 sequences make or modify molecules that affect the host. These include toxin synthases, enzymes that make capsules rendering bacteria resistant to phagocytosis, and “passive immune evasion” enzymes which alter microbial molecules to protect the possessor from host recognition and/or immune effectors. Examples of the latter include AlmG, a peripheral membrane aminoacyl transferase from V. cholerae that modifies lipopolysaccharide to resist host cationic antimicrobial peptides (279), and Cbu0678 from C. burnetii, which changes the O antigen of lipopolysaccharide (LPS) to decrease immune recognition (280).
3. Type 3 sequences are secretion system components that transport directly acting SoCs to the correct location for function. These include chaperones for the effector proteins.
4. Type 4 sequences are transcription factors regulating the expression of sequences that produce effects directly. While they can be very important for the virulence of a microbe and greatly influence how pathogenic a specific microorganism can be, they might be replaced in a GoF scenario by similar factors.

### (iii) What host cellular process is affected?

We found it helpful to annotate SoCs with the host processes that they modulate, as these can often be discerned before the biochemical mechanisms are discovered. No fewer than nine aspects of eukaryotic host cell biology are targeted by parasite proteins for manipulation: (1) transcription, (2) translation, (3) the cell cycle, (4) apoptosis, (5) ubiquitination, (6) small GTPase dynamics, (7) cytoskeleton dynamics, (8) endomembrane, dynamics, and (9) autophagy/xenophagy. Viruses tend to manipulate the first five processes, while bacteria, particularly intracellular parasites, affect the final six, with overlap at apoptosis and ubiquitination.

## DISCUSSION

Gauging the risks of an emerging pathogen strain or one created through microbial engineering (accidental or otherwise) requires a good comprehension of the pathogenic possibilities of SoCs from natural parasites of humans and livestock. An assessment of existing controlled vocabularies revealed a gap for sequences from nonviral parasites. We documented the role played in disease of over 2,750 parasite proteins from thousands of papers. These were annotated with the FunSoC schema, which categorizes their host-affecting features. The 220 sequences mentioned in this text are provided with full annotations in Data Set S2 in the supplemental material, with definitions provided in Data Set S1.

FunSoCs are tidy enough for human comprehension. For a given SoC, they provide a quick assessment for ∼30 host-affecting functions. However, they are insufficiently granular for capturing the molecular details necessary for a comprehensive appreciation of function. We think that these details are better understood with a new adjunct to GO, Pathogen Gene Ontology (PathGO). This resource is being developed by a group at the Johns Hopkins University Applied Physics Laboratory and consists of ∼180 terms (https://github.com/jhuapl-bio/pathogenesis-gene-ontology). These are being rooted in biological process and molecular function terms of the Gene Ontology resource (281, 282). We have been suggesting terms and contributing annotations during development. Data Set S2 features a preview of PathGO terms in column F, along with the relevant PubMed ID accession numbers as citations. PathGO will be described in a future publication.

### (i) The utility of gain-of-function experiments in microbial pathogenesis.

Sometimes eliminating a bacterial sequence suspected of involvement in pathogenicity has no effect. Legionella pneumophila exhibits so much functional redundancy in its effectors that the loss of one or two sequences of a certain type may not affect the phenotype (283). Investigators of bacterial adhesion face a similar situation when the suspected adhesin originates in a microbe with multiple ways of associating with a target cell. Researchers circumvent this by studying the adhesin in the background of a specially selected “nonadherent” bacterium (284–289). Experiments in which a sequence “adds” virulence to commensals or avirulent microbes is more interpretable than attempts to ascertain virulence by subtraction from a pathogenic background. The former involves a GoF for the avirulent microbe.

Only a few efforts to make bad bugs worse intentionally have been described (290). However, there are hundreds of publications relating the expression of one or more sequences from an infectious parasite in a heterologous organism. Two dozen of these are noted in column E of Data Set S2. Altered organisms typically display a new property consistent with the suspected pathogenic function of the sequence in the original organism. These GoF experiments are illuminating but can also be problematic (291, 292). The role that a sequence plays in the pathogenicity of a microbe can depend on other proteins and/or the timing of its expression. Simply expressing the sequence in another microbe, even a similar one, is no guarantee that it will perform similarly. The question can be settled only empirically within the limits of the model. The most dramatic example of a GoF experiment with biothreat implications is the notorious mouse interleukin-4 (IL-4) expression in Ectromelia virus that was astoundingly lethal in even vaccinated animals (293). An intriguing bacterial example involves the secreted protease SpyCEP of group A Streptococcus. When the nontoxic SpyCEP was expressed in the nonpathogenic bacterium Lactococcus lactis, it rendered the cheese-making firmicute capable of infection in a mouse leg wound model. The SpyCEP protease degrades the chemokine interleukin-8, which host neutrophils use to coordinate their defense, “sniffing out” bacteria within infected tissues. Interruption of this coordination produced a systemic disease that had lethal consequences for the host within 24 h of inoculation (230).

### (ii) Recognized criteria for sequences of concern improve biosecurity.

For those worried about either the accidental engineering of pathogens via synthetic biology or the production of bioweapons with enhanced efficacy, a concerning sequence is one that, when transferred to a different microbe, increases the ability of that microbe to damage a susceptible host, increasing the pathological consequences of infection. But, as the cases of SpyCEP and murine IL-4 demonstrate, the disease-causing properties of microbes have interesting dependencies that cannot be understood in the absence of experiments. We think that the criterion of enhanced pathogenicity upon expression in a heterologous nonpathogen is a good starting place for identifying SoCs, but most will not be discovered through such GoF experiments. Our annotation project has demonstrated that there are thousands of microbial sequences that can reasonably be assumed to enhance the pathogenic ability of a heterologous microbe if transferred. In such cases, the disease-causing properties of these sequences are described in the context of the original pathogenic organism and not in a heterologous, nonpathogenic microbe. We assume that these sequences may retain their properties if transferred to a similar microbe. At the very least, it does not seem responsible to assume that they would be innocuous. Documenting these sequences enables them to be recognized via bioinformatics and thus improves biosecurity for those involved in the manufacture of synthetic nucleic acids (2).

Toxins and microbial effectors that damage the human host are of greatest concern. Among these, SoCs that provoke organ failure have the most severe consequences. Next in importance are sequences that subvert host immunity. Noting the host cellular process(es) with which a SoC interacts and how directly it affects host molecules allows a better understanding of its role in microbial pathogenesis. Formalizing these criteria improve recognition of SoCs from the literature, provide the means for distinguishing them by function, and permit the reporting of these functions in bioinformatic applications. We think that the FunSoC vocabulary and data sets annotated with it can be a resource for computational epidemiology, microbial genomics and forensics, DNA synthesis screening, human disease modeling, and biosecurity assessment.
